# Moving beyond dosage and adherence: A protocol for capturing dimensions of active child engagement as a measure of fidelity for social-emotional learning interventions

**DOI:** 10.3389/fpsyg.2022.1014713

**Published:** 2023-01-09

**Authors:** Brianna L. Devlin, Tanya M. Paes, Elyssa A. Geer, Lindsey M. Bryant, Tracy M. Zehner, Irem Korucu, Kathleen Morse, Robert J. Duncan, David J. Purpura, Sara A. Schmitt

**Affiliations:** ^1^Human Development and Family Studies, Purdue University, West Lafayette, IN, United States; ^2^Yale Center for Emotional Intelligence, School of Medicine, Yale University, New Haven, CT, United States

**Keywords:** social-emotional learning (SEL), social-emotional learning interventions, implementation, child engagement, participant responsiveness, preschool

## Abstract

Social-emotional competencies are important for school-readiness and can be supported through social-emotional learning (SEL) interventions in the preschool years. However, past research has demonstrated mixed efficacy of early SEL interventions across varied samples, highlighting a need to unpack the black box of which early interventions work, under what conditions, and for whom. In the present article we discuss the critical implementation component of active child engagement in an intervention as a potential point of disconnect between the intervention as designed and as implemented. Children who are physically present but unengaged during an intervention may lead to decreased average impacts of an intervention. Furthermore, measuring young children’s active engagement with an intervention may help to guide iterative intervention development. We propose a four-step protocol for capturing the multi-dimensional and varied construct of active child engagement in a SEL intervention. To illustrate the utility of the protocol, we apply it to data from a pilot study of a researcher-implemented, semi-structured block play intervention focused on supporting the development of SEL and math skills in preschoolers. We then present future directions for the integration of active participant engagement into the measurement of implementation of SEL interventions for young children.

## 1. Introduction

Social-emotional competencies, including getting along with others, paying attention to and following directions, and regulating behaviors and emotions are crucial skills for children to develop prior to school entry as they predict later academic achievement and well-being (McClelland et al., 2007, 2013; Schmitt et al., 2017; Mackintosh and Rowe, 2021). However, interventions targeting social-emotional competencies in preschool have had mixed success, and scholars have recently suggested this may be due to issues with implementation fidelity, or perhaps, the way fidelity is typically measured (McClelland et al., 2017). For example, in the large majority of studies, implementation fidelity is typically assessed using simple measures of dosage (i.e., “Did they do it?”) and adherence (i.e., “Did it align with the guidelines?”). Active participant engagement (i.e., participation in the intervention) is seldom measured despite its inclusion in theoretical models of implementation (Century et al., 2010; Berkel et al., 2011). When active participant engagement in SEL interventions is included as a measure of fidelity, it almost exclusively concerns the active engagement of adults (e.g., teachers) with the intervention, and not child participant engagement.

Discounting how the autonomy of young children could lead to individual differences in children’s active participation during an intervention may lead to biased estimates of the efficacy of early SEL interventions. That is, a child may be physically present at an intervention session, but not actively engaging with the target material. Moreover, they may actively engage with only specific aspects or activities of the intervention but not others. These differences would not be captured by traditional measures of child-level dosage and could result in diminished effectiveness of an intervention as measured by estimates of average impacts. By assessing active child engagement as a multi-dimensional construct of implementation fidelity, we may be better able to capture individual differences in children’s experiences that moderate the effect of an intervention. Furthermore, considering the nuanced ways in which young children engage with an intervention may help researchers to iteratively develop interventions that support children from diverse backgrounds (e.g., considering differences by socio-cultural groups, site specific needs, etc.). Consequently, in the present article, we introduce a four-step protocol developed to capture active child engagement with SEL interventions. We use data from a pilot study of a semi-structured block play intervention (Schmitt et al., 2018a) as an example of applying the protocol to capture dimensions of active child engagement.

### 1.1. Supporting children’s early social-emotional learning through targeted interventions

Our broad definition of SEL interventions include intervention programs aimed at supporting the development of social skills, emotion regulation, and cognitive regulation (i.e., executive functions; McClelland et al., 2017). This definition is in line with the Collaborative for Academic Social and Emotional Learning (CASEL)’s framework that emphasizes the importance of competencies spanning social, emotional, and cognitive regulatory processes (CASEL, 2017). Several SEL interventions have been created to bolster these competencies in early childhood (e.g., McClelland et al., 2017; Nesbitt and Farran, 2021), a critical period in which rapid changes occur in cortical brain structures that are vital for SEL and cognitive development (Garon et al., 2008). This developmental period also coincides with children engaging with adults and peers in multiple settings (e.g., home, preschool; Schmitt et al., 2018a), in which SEL skills are necessary for successful relationships.

A common theme across the majority of early SEL interventions is that they are child-centered, which is thought to promote active child engagement with intervention content (Massouleh et al., 2012). For example, the Red Light, Purple Light intervention (Tominey and McClelland, 2011; Schmitt et al., 2015; McClelland et al., 2019) centers children’s experiences and active engagement by using fun and age-appropriate music and movement activities designed to promote behavioral self-regulation. During this intervention, children are given agency in interacting with the games as they choose and are also offered opportunities for autonomy in leading games. As another example, the Preschool Alternative THinking Strategies (PATHS) program offers explicit instruction in SEL through teacher-led lessons and extension activities like group games and art projects (Domitrovich et al., 2007). These activities were designed to be fun and engaging for young children while also promoting their SEL skills broadly. Brain Games (Barnes et al., 2021) is another classroom-based intervention for young children, focused on building attention, working memory, and inhibitory control through games.

Despite the fact that many SEL interventions take a child-centered approach, evidence of the efficacy of these interventions is mixed (McClelland et al., 2017; Nesbitt and Farran, 2021). Although some interventions like Red Light, Purple Light (Tominey and McClelland, 2011; Schmitt et al., 2015) and PATHS (Domitrovich et al., 2007) have shown positive effects on their target outcomes in preschoolers, other programs have shown mixed or null effects. For example, in a recent study Nesbitt and Farran (2021) found no positive impacts of the Tools of the Mind curriculum for supporting SEL in early childhood. Of note, Nesbitt and Farran were not the original developers of the Tools of the Mind curriculum. When researchers analyzed the implementation fidelity factors of adherence and dosage they found that intervention classrooms had varied dosage but implemented about half of what the developers expected, on average. However, neither the adherence to nor amount of time spent on the Tools curriculum was statistically related to children’s outcomes.

### 1.2. Integrated models of intervention implementation

To unpack the nuances of which early SEL interventions work under which conditions and for whom, it is essential to consider multiple factors of implementation fidelity. Theoretical frameworks of program implementation include factors controlled by the intervention designers (e.g., differentiation of intervention practices from currently enacted practices), factors controlled by the interventionist (e.g., script adherence and quality of delivery), and factors controlled by the participant (e.g., attendance and active engagement; Dane and Schneider, 1998; Carroll et al., 2007; Berkel et al., 2011). For example, Berkel et al.’s (2011) integrated model of program implementation differentiated behaviors that occur at the time of implementation that are directed by the interventionist from those directed by the participant (i.e., participant responsiveness). Furthermore, interventions can sometimes include multiple levels of implementation, such as when researchers train providers, who go on to train teachers or parents, who then teach children. Responsiveness to the intervention as intended includes attendance and dosage, retention, satisfaction, and active participant engagement, or participation in an intervention (Berkel et al., 2011). However, reviews of implemented educational interventions have demonstrated that interventionist-controlled factors (especially adherence) are more commonly reported than measures of participant responsiveness (O’Donnell, 2008). When responsiveness is considered, dosage is the most often-reported measure (Bos et al., 2022). Measurement of active participant engagement in early childhood interventions has primarily been concentrated on the adult participants who implement the intervention with children (e.g., teachers and parents’ active participation with the intervention). Some work has started to unpack the complex relations among fidelity indicators, including active participant engagement, at differing levels of an intervention. For example, Berkel et al. (2018) tested a theoretical cascade model in which facilitator delivery (e.g., adherence of the provider) predicted participant responsiveness (e.g., parents’ home practice), which in turn lead to improvements in the targeted outcomes (e.g., children’s mental health). However, very few studies have considered participant responsiveness fidelity indicators, such as active participant engagement, at the child-level of interventions designed to improve outcomes for young children.

## 2. Capturing dimensions of active child engagement

Given the lack of studies that report active child engagement—especially for young children—we sought to develop a general protocol for capturing young children’s active engagement with interventions as a measure of fidelity. The development of this protocol was guided by a conceptual framework comprised of the following assumptions:

• Individual differences in active child engagement may influence intervention efficacy.
• Contextual and individual factors may shape differences in active child engagement with an intervention.
• Active child engagement with an intervention can be measured through observing behavior.
• There are multiple dimensions to active child engagement with an intervention.
• There is variability in active child engagement with an intervention.

### 2.1. Individual differences in active child engagement may influence intervention efficacy

Active child engagement occurs within each intervention session, and thus “serves as a potential source of disconnect between the program as intended and the program as implemented” (Berkel et al., 2011, p. 23), making it a worthwhile focus of effort for measuring implementation fidelity. Dosage is a commonly reported measure of children’s participation in an intervention, but this fidelity indicator does not provide information about whether children experienced the intervention as it was intended. That is, children who are physically present but unengaged during intervention sessions may lead to decreased efficacy of the intervention as measured by estimates of average impacts. Therefore, it is important to consider how active child engagement may moderate the effect of an intervention.

### 2.2. Contextual and individual factors may shape differences in active child engagement with an intervention

Beyond considering how individual differences in engagement may influence intervention efficacy, it is also important to consider what factors predict differences in engagement with an intervention. School level factors such as discipline policies or classroom-level factors such as classroom culture and norms may affect active child engagement with a SEL intervention. For example, when schools and centers enact disciplinary measures that involve pulling children from an activity or classroom, they increase the chances of disrupting active child engagement. Classroom factors like the number of children in play groups have been associated with children’s positive engagement with peers, as smaller groupings promote more cooperative play (Howes et al., 2011). Intervention factors such as who is implementing the intervention (e.g., teachers or researchers) may also influence active child engagement. Finally, child level factors such as demographic background and baseline skills may influence children’s active engagement. By conducting in depth analyses of how children engage with an intervention as a measure of responsiveness, we can gather information that may challenge researchers’ assumptions about how children respond to materials and prompts. This is an especially important undertaking when working with historically under-represented populations in past research. This information may be used to inform iterative development of the intervention in an effort to create interventions that promote active child engagement for children from diverse backgrounds.

### 2.3. Active child engagement with an intervention can be measured through observing behavior

Although most research focused on active participant engagement has utilized participant report with adult participants (Carroll et al., 2007), engagement can be assessed through observing behavior, opening the door for assessment of engagement in young children. For example, Ling and Barnett (2013) used behavior observations time-sampled in 15-s intervals to assess groups of preschoolers’ engagement in circle time activities. Another researcher-developed measure is the inCLASS (Downer et al., 2010), which is an observational tool focused on capturing preschoolers’ interactions with teachers, peers, and classroom activities. In the context of early SEL interventions, Schmitt et al. (2018b) used a similar measure to assess preschoolers’ on-task behavior during the Positive Action intervention, aimed at improving social-emotional competence and health behaviors. One efficacy study of the Tools of the Mind intervention also included a measure of child engagement with the intervention using a Likert scale based on observations of child behavior ranging from completely off-task to intense focus across time-sampled intervals (Child Observation in Preschool, COP; Nesbitt and Farran, 2021).

### 2.4. There are multiple dimensions to active child engagement with an intervention

The aforementioned work that measured and reported active child engagement in interventions approached it as a unidimensional construct and did not consider the complex nature of the intervention environment in which young children often have varied points of opportunity for engagement with an intervention as intended. For example, a child may appear to be actively engaged in an intervention activity (e.g., by being socially engaged with their peers) without engaging with the targeted SEL content. As another example, in a randomized trial of the Red Light, Purple Light intervention, the authors describe that although “the majority of children actively participated in all of the playgroup games… a few children chose to watch on occasion” (Tominey and McClelland, 2011 p. 513). Although these children were actively engaged with the content of the games by watching, they were not actively engaged with the physical movements of the games, a separate dimension of engagement. By considering active child engagement as a unidimensional construct, we miss capturing varied levels of engagement with the intervention, which may provide biased estimates of program outcomes. Furthermore, considering multiple dimensions of active child engagement allow for the ability to test which child participant-involved components are most important for growth in the target outcomes. Thus, a key extension of existing research is our consideration of active child engagement with an intervention as a multi-dimensional construct.

### 2.5. There is variability in active child engagement with an intervention

A final assumption behind our approach to measuring active child engagement is that it is varied, both between and within children. Decades of research in cognitive developmental psychology have converged to support the theory that development is defined by variability (Siegler, 2007). That is, differences in children’s thinking and behavior exist between individual children, within the same child in different contexts, and even within the same child in the same context at a different point in time (Siegler, 1994). We believe that it is important to take this variability into account when approaching the measurement of active child engagement with an intervention. For example, a child’s engagement may vary across intervention sessions in response to the content, or across the course of a single session. By operationalizing engagement in a way that takes variability into account, researchers can capture nuances such as the “implementation dip,” or a decrease in performance or adherence in response to change (Fullan, 2001) in the context of active child engagement with SEL interventions.

## 3. Four-step protocol for capturing dimensions of active child engagement

In line with past theoretical frameworks that use a multi-step approach as a solution for measuring the complex construct of fidelity (e.g., Hulleman et al., 2013), we propose a four-step protocol for capturing active child engagement:

1. Identify points of opportunity for active child engagement with the intervention to specify multiple dimensions of active child engagement.
2. Operationalize and measure the dimensions of active child engagement.
3. Analyze the dimensions of active child engagement.
4. Link the dimensions of active child engagement to other variables.

### 3.1. Applying the four-step protocol to measure preschoolers’ active engagement with the Block Play Intervention

In this section, we use data from a pilot study of the Block Play Intervention to illustrate application of the four-step protocol. The Block Play Intervention is a brief, semi-structured, play-based intervention aimed at supporting preschoolers’ cognitive regulation, a critical component of SEL (McClelland et al., 2017), and mathematics through small group interactions. The intervention includes twice-weekly sessions of small group play (two to three children) with wooden unit blocks. Children are given specific building goals at the start of the session by a researcher interventionist (e.g., *“Today your job is to build a tower together!”*) but are then allowed to build freely. Over the course of the intervention sessions, the prompts gradually become more complex so that children’s cognitive regulation is challenged, not just used (e.g., Week five: *“Today your job is to build a house together*… *It needs to have four walls, a roof, a way to get inside like a door, and at least two rooms*”; Week seven: *“Today I am going to show you a picture of a structure. Your job is to work together to build the structure you see in this picture.”*). These prompts were targeted at priming children to work together and engage in social interaction to build structures in collaboration with their groupmates while also building their self-regulation skills through avoiding distractions and engaging in goal-oriented behavior.

**FIGURE 1:**
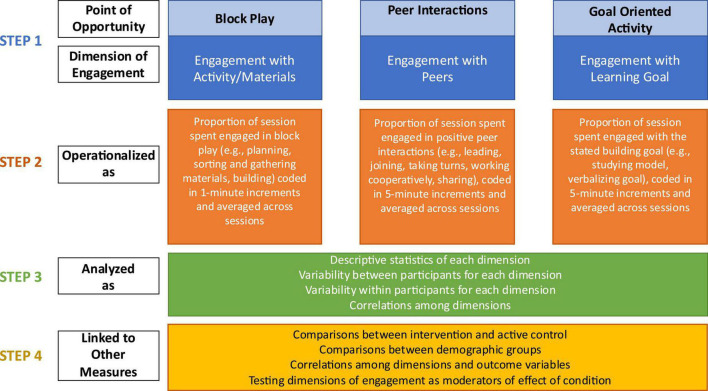
Applying the four-step protocol to measure preschoolers’ active engagement with the Block Play Intervention.

A pilot study of this intervention included a sample of 59 children (*M*_age_ = 55.20 months), randomly assigned to the intervention group (*n* = 24) or a business as usual (BAU; *n* = 35) control group. On average, children in the intervention condition participated in 13 of the 14 sessions, with a range of 11 to 14 sessions attended. Researcher interventionists adhered to the building prompt scripts 94% of the time. The application of the four-step protocol for capturing active child engagement to the pilot data of the Block Play Intervention is presented visually in Figure 1. We use the context of this researcher-implemented pilot study as a straightforward example of the protocol’s application but believe the four-step protocol can be scaled and utilized with much larger and more complex SEL interventions with young children.

#### 3.1.1. Step 1: Identify points of opportunity for engagement to specify dimensions of active child engagement

**FIGURE 2:**
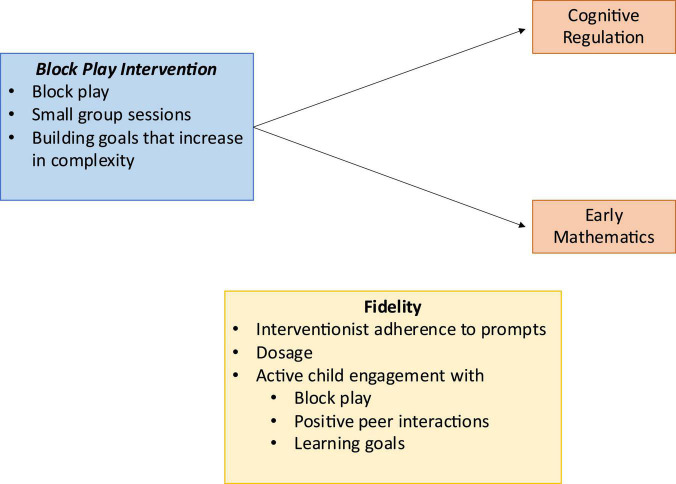
Theory of change for the Block Play Intervention.

The first step is to identify opportunities for potential active child engagement with the intervention. As with all aspects of fidelity, the identification of active child engagement opportunities should be closely tied to the intervention’s overarching theory of change (Darrow, 2013). It may be helpful to think of these points of opportunity as participant-involved core intervention components, or the aspects of the intervention in which child participants are directly involved that are theorized to lead to change in the child-level outcome variables. The theory of change of the Block Play Intervention is presented in Figure 2.

In the Block Play Intervention, we hypothesized that participation in semi-structured block play sessions would lead to gains in the outcomes of interest through three points of potential active child engagement: the block play itself, positive interactions with peers, and working toward a provided, increasingly complex goal (see Row 1 of Figure 1). Block play provides children with opportunities to practice working with abstract concepts and representations, which may help to develop cognitive regulation (Wolfgang et al., 2001; Hadani and Rood, 2018). Children also get the opportunity to practice fine motor skills through block play, which relate to cognitive regulation and mathematical cognition development in the early years (Gashaj et al., 2019; McClelland and Cameron, 2019). Working cooperatively with peers by negotiating and engaging in prosocial behaviors may help children to develop their language and interpersonal skills (Sluss and Stremmel, 2004), as well as emotional and behavioral self-regulation skills like inhibiting prepotent responses (e.g., knocking down a peers’ tower when angry). Finally, the goal-directed aspect of the activity may also help to strengthen children’s cognitive regulation and academic skills as children are required to utilize metacognitive resources to remember and plan their building behavior in accordance with the goal (Schmitt et al., 2018a).

After identifying these points of opportunity for potential engagement, we used them to generalize and name the multiple dimensions of participant engagement that we wished to capture. For the Block Play Intervention, this resulted in three general dimensions of child engagement: Engagement with the activity/materials, engagement with peers, and engagement with the learning goal (see Row 2 of Figure 1). Although our specified dimensions may apply to many play-based or semi-structured early SEL interventions, researchers wishing to use this protocol can alter or substitute these dimensions as needed. For example, SEL interventions that include teacher or parent interactions may need to add a dimension for children’s engagement with a caregiver. Furthermore, researchers testing more comprehensive interventions may wish to draw the points of opportunity for potential engagement from the specific activities in the intervention or create multiple levels (e.g., engagement with materials and peers within specific intervention activities like shared book reading, role playing, or group games).

#### 3.1.2. Step 2: Operationalize and measure dimensions of active child engagement

The second step is to operationalize each dimension of active child engagement (see Row 3 in Figure 1) so that it can be quantified. Measurement decisions should be guided by pilot work, past literature, and the intervention theory of change. Researchers may choose to use previously developed measures of child engagement (e.g., inCLASS, Downer et al., 2010; COP, Farran, 2011) or to develop their own observational measures of specific behaviors.

##### 3.1.2.1. Engagement with the activity/materials in the Block Play Intervention

Aligned with our conceptual framework, we chose to operationalize each dimension of child engagement with the Block Play Intervention through observation of behavior captured on video recordings of the 15-min block play sessions. For the engagement with activity/materials dimension, we used time-sampled observations of “on-task” behavior (Ling and Barnett, 2013). Coders watched videos and documented codes for each child individually, replaying sections of the video as needed. As past pilot work suggested that children would engage in on-task block play behavior for much of the session, we chose to use momentary time-sampling of whether or not children were actively engaged in on-task block play behavior after every minute of play. That is, it was important to use a higher frequency of coding instances (after every minute) to adequately capture variability and examine individual differences in children’s engagement with the activity and materials. Children received a score of 0–15 for each session.

##### 3.1.2.2 Engagement with peers in the Block Play Intervention

When operationalizing the engagement with peers dimension, we chose to focus on positive peer interaction behaviors as they were hypothesized to be related to gains in the outcome variables. We drew the positive peer interaction behaviors of interest from two sub-scales of the Minnesota Preschool Affect Checklist Revised/Shortened (M-PAC-R/S; Denham et al., 2012)–the Leading and Joining and the Empathy and Prosocial Behavior sub-scales. We coded in 5-min whole-interval time-sampled increments (a coarser level of analysis than the engagement with activity/materials dimension) in line with the protocol for using the M-PAC-R/S. Children therefore received a score of 0–3 for each session corresponding to positively engaging with peers during none of the three 5-min intervals (0) to positively engaging with peers during all of the three 5-min intervals (3). To code, we determined if a child engaged in any of these five behaviors at any time in the prior 5 min: successfully leading an activity, successfully joining an activity, facilitating turn-taking, cooperating with a peer or group to achieve a common goal, or exhibiting sharing behavior. For example, consider this dyad of children who were given the goal of building a castle for a king and queen. In the process, they engaged in leading and joining behaviors, taking turns in adding blocks to the structure, and working together cooperatively:

Child A: [Pointing to blocks] “*So this is the king, that’s the queen. King, queen. And we need a bottom slip. That will help*… *So that will go right here.*”
  Child B: “*Put it right on this.”*
  Child A: “*We need another one of these pieces.”*
  Child B: “… *I found one!”*

A different dyad of children took another cooperative building approach, where one child led the building activity, and another gathered and shared materials:

Child C: “*Wait, wait*… *I need baby triangles*… *I need another triangle*… *A baby one. Will you find it for me?”*
  Child D*: “I will find it for you.”*
  Child C: [taking triangle from Child D] “*This goes here.”*

##### 3.1.2.3 Engagement with learning goals in the Block Play Intervention

Finally, we operationalized engagement with the learning goal as the proportion of the session a child spent engaged with the explicit building goal given to them by the interventionist, as evidenced by verbal and non-verbal behaviors. This dimension was also coded in whole-interval 5-min increments by determining whether a child spent any of the time in the previous 5 min building the assigned structure for the session. Children therefore received a score of 0–3 for each session corresponding to engaging with the building goal during none of the three 5-min intervals (0) to engaging with the building goal during all of the three 5-min intervals (3). Examples of children who did not engage with the building goal varied. For example, when asked to build a tower, one child responded, *“No thank you. We are making a playground.”* Other children chose not to engage with the given building goal, and instead requested to be able to build freely. For example, when children were asked to model their structure after a picture, one child responded, *“I’m not gonna build that. Can I just build?”*

For each dimension of engagement, we calculated the proportion of the session spent engaged in the specified behaviors of that dimension. That is, for the engagement with activity/materials dimension, we calculated the proportion of minutes children were engaged in on-task block play behavior out of the total 15 min. For the engagement with peers and learning goals dimensions, we calculated the proportion of intervals children were engaged in either leading, joining, taking turns, working cooperatively and sharing behaviors or goal-oriented behaviors out of the total three coded intervals. Proportions were averaged across the 14 sessions to create an overall engagement score for each dimension throughout the course of the intervention. As we were also interested in considering within-session variability, we also averaged across sessions to create a variable of average engagement in each dimension for the first 5 min, middle 5 min, and final 5 min of the 15-min sessions.

#### 3.1.3. Step 3: Analyze dimensions of active child engagement

The third step of the protocol is to intensively analyze the dimensions of engagement variables. The overarching goal of this step is to answer the questions of whether children actively engaged in the intervention as intended across dimensions, and how their engagement in each dimension varied within and across sessions. See Row 4 of Figure 1 (Step 3) for suggestions on how to analyze these variables.

##### 3.1.3.1. Engagement with the activity/materials in the Block Play Intervention

**FIGURE 3:**
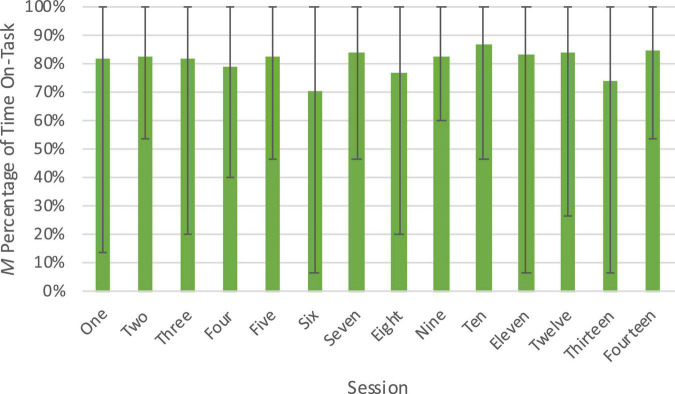
Engagement with the activity/materials across sessions of the Block Play Intervention. Figure shows percentage of engagement out of total coded time (15 min) with the activity/materials at each session. The range from minimum to maximum engagement is represented by error bars.

Data was converted to percentages of the calculated proportions for interpretation, but descriptive statistics of the raw data by session are presented in Table 1. Children in the intervention condition were engaged with on-task block building activity 81.0% of the time on average. There was variability between individuals, with the least engaged child engaging in on-task behavior an average of 58.5% of the time and the most engaged child engaging in on-task behavior an average of 95.2% of the time. Examining within-individual variability also provided valuable information about whether the design of the intervention was able to sustain children’s engagement in on-task block play over time. Children were fairly consistent with their engagement over the course of the 15-min sessions, although there was some decline; they engaged in on-task behavior 84.4% of the time, on average, in the first 5 min of sessions, 81.4% of the time in the next 5 min, and 73.6% of the time in the last 5 min. There was also some variability in engagement with the activity/materials across sessions (see Figure 3), but there was not an overall decline in engagement with block play as the intervention went on. Overall, our analysis of this dimension suggests that although on-task behavior was high, there was between-individual variability which could potentially influence the strength of efficacy of the intervention. Furthermore, our design of the block play sessions seems to have been successful at keeping children actively engaged in on-task behavior across the intervention sessions.

**TABLE 1:** Descriptive statistics by session.

Dimension of engagement		Session number													
		**1**	**2**	**3**	**4**	**5**	**6**	**7**	**8**	**9**	**10**	**11**	**12**	**13**	**14**
**Activity/Materials** 0–15 1-min intervals	*M*	12.3	12.4	12.3	11.9	12.4	10.5	12.6	11.6	12.3	13.0	12.5	12.6	11.1	12.7
	*SD*	3.1	2.3	3.3	2.6	2.1	4.7	2.4	3.4	2.3	2.0	3.2	3.1	3.8	2.2
	Min	2.0	8.0	3.0	6.0	7.0	1.0	7.0	3.0	9.0	7.0	1.0	4.0	1.0	8.0
	Max	15.0	15.0	15.0	15.0	15.0	15.0	15.0	15.0	15.0	15.0	15.0	15.0	15.0	15.0
**Peers** 0–3 5-min intervals	*M*	1.9	2.0	2.2	1.7	2.0	1.4	1.8	1.2	1.5	0.9	2.3	1.8	1.5	2.1
	*SD*	1.1	1.2	0.9	1.2	1.1	1.2	1.3	1.2	1.2	1.0	0.9	1.3	1.1	0.9
	Min	0.0	0.0	0.0	0.0	0.0	0.0	0.0	0.0	0.0	0.0	0.0	0.0	0.0	0.0
	Max	3.0	3.0	3.0	3.0	3.0	3.0	3.0	3.0	3.0	3.0	3.0	3.0	3.0	3.0
**Learning goals** 0–3 5-min intervals	*M*	2.4	2.5	1.9	2.3	2.6	2.2	2.4	2.4	2.5	2.5	2.2	2.1	2.0	2.0
	*SD*	0.9	0.9	1.4	1.1	0.7	0.6	0.6	0.9	0.8	0.5	1.1	1.0	1.1	1.0
	Min	0.0	0.0	0.0	0.0	1.0	1.0	1.0	0.0	0.0	2.0	0.0	0.0	0.0	0.0
	Max	3.0	3.0	3.0	3.0	3.0	3.0	3.0	3.0	3.0	3.0	3.0	3.0	3.0	3.0

##### 3.1.3.2. Engagement with peers in the Block Play Intervention

**FIGURE 4:**
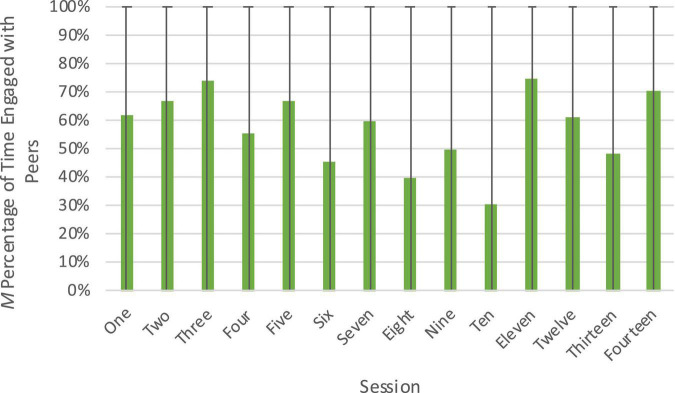
Engagement with peers across sessions of the Block Play Intervention. Figure shows percentage of engagement out of total coded time (three time intervals) with peers at each session. The range from minimum to maximum engagement is represented by error bars.

Descriptive statistics of the raw data by session are presented in Table 1. Children were engaged in positive peer interactions an average of 57.0% of the intervals sampled. There was substantial variability between individual children, with the least engaged child only engaged in positive interactions with peers an average of 12.0% of the time, and the most engaged child engaged an average of 97.7% of the time. Children were most engaged in positive interactions with peers near the start of the session (61.5% in the first 5 min, on average), which declined over the course of the session (54.2% in the next 5 min and 50.0% in the last 5 min, on average). There was also considerable variability in children’s engagement with peers across sessions (see Figure 4). However, there was not an overall pattern of children positively engaging with peers more or less as the intervention went on.

Overall, analyses of the Engagement with Peers dimension highlighted that despite the prompts for children to work together on their building, children only positively engaged with peers an average of 57% of the sampled intervals. As cooperative play typically emerges between 4 and 5 years of age (Parten, 1932), this level of peer interaction seems developmentally appropriate. Children were more likely to engage positively with peers just after the building prompt was given (in the first 5 min), suggesting that adding scaffolding of peer interactions or reminders to work together throughout the course of the sessions in future iterations of the intervention may increase positive peer engagement.

##### 3.1.3.3 Engagement with learning goals in the Block Play Intervention

**FIGURE 5:**
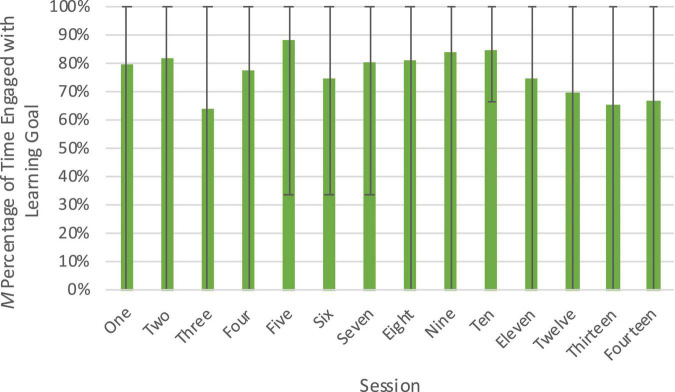
Engagement with learning goals across sessions of the Block Play Intervention. Figure shows percentage of engagement out of total coded time (three time intervals) with the learning goal at each session. The range from minimum to maximum engagement is represented by error bars.

Descriptive statistics of the raw data by session are presented in Table 1. Children were engaged with the provided building goal an average of 77.0% of the intervals. There was variability between the least engaged child (39%, on average) and the most engaged child (100%). Again, children’s engagement with the goal generally declined over the course of the session, with a drop-off near the end of the sessions (*M* = 84.3% in first 5 min; 80.0% in second 5 min; 63.9% in the last 5 min). There was some variability in goal engagement across the course of the intervention (see Figure 5). With the exception of one session early on, engagement with the goal was lowest for the most complex building goals, in which children were required to copy a model of a sophisticated structure, given in the final four sessions.

##### 3.1.3.4. Engagement across dimensions in the Block Play Intervention

**FIGURE 6:**
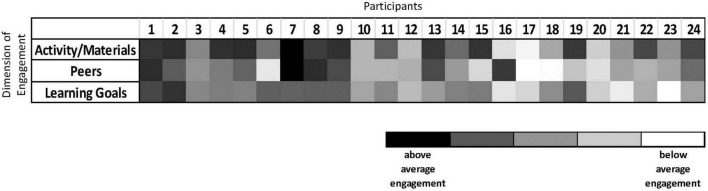
Heat map of individual children’s multi-dimensional active engagement with the Block Play Intervention. Figure shows individual children’s engagement with activity/materials, peers, and learning goals across the Block Play Intervention. Each column represents an individual child, and the rows illustrate that child’s engagement with a specific dimension, relative to other children in the sample. Darker shades demonstrate that a child was increasingly above average, compared to the rest of the sample, on a dimension of engagement. Lighter shades demonstrate that a child was increasingly below average on a dimension of engagement.

To demonstrate multi-dimensionality of engagement with the Block Play Intervention for the 24 children in the pilot intervention condition, we standardized the dimensions of engagement variables and have presented them as a heat map in Figure 6. Each column represents an individual child, with increasingly above average engagement in a specific dimension represented as a darkening shade. Below average engagement is indicated with lighter shades. Some children were uniformly engaged or disengaged with the intervention across the dimensions. For example, Participant 1 was above the average of the sample in their engagement with the activity/materials, with peers, and with the learning goal. Conversely, Participant 17 was below average in their engagement across all dimensions. However, other children showed more nuanced engagement with different aspects of the intervention. Participant 16 was highly positively engaged with their peers compared to the rest of the sample but was below average in their engagement with the activity and the goal. Specifically, this child was frequently distracted from building (e.g., walking around the room) and when they did engage in building, they preferred to set their own goals. However, they were highly social with peers, offering positive encouragement and engaging in sharing behaviors during play. Participant 19 was engaged with block play activity in alignment with the provided building goal but was not as positively engaged with peers as the rest of the sample, as this child preferred to build alone. When a peer did attempt to engage with them, the interactions were often negative (e.g., refusing to let another child help with the structure). Finally, Participant 13 was highly engaged with block play and with peers compared to the rest of the sample but was slightly below average in their engagement with the provided building goal. These results demonstrate that, even in this small sample, children were differentially engaged with the multiple points of opportunity for engagement. By separating the multiple dimensions of engagement, we can test the most important dimensions of engagement for intervention efficacy in the fourth step of the protocol.

#### 3.1.4. Step 4: Link active child engagement to other measures

In the final step of the protocol, the dimensions of active child engagement should be linked to other variables. First, researchers can explore the assumption that differences in active child engagement can be shaped by contextual and individual factors, like school-level, demographic or baseline skill variables. Next, it is important to link the dimensions of active child engagement to outcome measures to assess how active child engagement may influence intervention effectiveness.

##### 3.1.4.1 Individual predictors of active child engagement in the Block Play Intervention

To consider individual-level predictors of active child engagement with the Block Play Intervention, we estimated correlations among the dimensions of engagement and the demographic variables of age and parent education level, as well as children’s baseline social skills and problem behaviors as rated by their classroom teacher using the Social Skills Improvement System rating scale (Gresham and Elliott, 2008; see Table 2). Teachers were asked to use the scale to rate children’s frequency of behaviors in social skills including communication and cooperation and problem behaviors including hyperactivity/inattention. We also calculated an overall engagement score by summing the standardized scores of engagement across each dimension. Results of these correlations should be interpreted cautiously, given the small sample size. Age was not significantly correlated with any dimension or overall engagement (all *p* values > 0.05). However, parent education was significantly correlated with overall engagement (*r* = 0.54, *p* = 0.01), engagement with activity/materials (*r* = 0.43, *p* = 0.04), and engagement with peers (*r* = 0.48, *p* = 0.02). Parent education and engagement with the learning goals were not significantly correlated at *r* = 0.31, *p* = 0.16.

**TABLE 2:** Descriptive statistics and correlations among dimensions of engagement and variables of interest for Step 4 of the protocol from the *Block Play Intervention*.

		Correlations			
	**Mean (*SD*)**	**Engagement with Activity/Materials**	**Engagement with Peers**	**Engagement with Learning Goals**	**Overall engagement**
Engagement with Activity/Materials	81% (11.9%)	-			
Engagement with Peers	57% (25.0%)	0.60**	-		
Engagement with Learning Goals	77% (17.3%)	0.77**	0.51**	-	
Overall engagement (*z-composite*)	0 (2.35)	0.89**	0.85**	0.86**	-
Age in months	57.6 (6.32)	0.11	-0.12	0.20	0.05
Parent education	5.61 (2.41)	0.43**	0.48**	0.31	0.54**
Social skills	2.07 (0.36)	0.46**	0.11	0.60**	0.40**
Problem behaviours	0.47 (0.35)	-0.56**	-0.26	-0.76**	-0.55**
Gain in behavioral self-regulation	18.8 (22.3)	0.18	0.19	0.17	0.20
Gain in cognitive flexibility	3.47 (5.18)	0.06	0.39*	-0.03	0.19
Gain in mathematical language	1.6 (1.8)	0.22	0.08	-0.04	0.07

**Indicates *p* < 0.05, *indicates *p* < 0.10; Parent education ranged from 8th grade or less (1) to doctoral degree (9). On average, parents had some college experience. Social skills and problem behaviors were measured by teacher rating scale (SSIS). Behavioral self-regulation was assessed using HTKS task, cognitive flexibility was assessed using DCCS task, and mathematical language was assessed using the math language assessment.

Teacher-ratings of children’s social skills were also significantly correlated with overall engagement (*r* = 0.40, *p* = 0.04), engagement with the activity/materials (*r* = 0.46, *p* = 0.03) and with the learning goals (*r* = 0.60, *p* = 0.002). Although the correlation between baseline social skills and engagement with peers was not statistically significant, it was positive (*r* = 0.11, *p* = 0.57). Conversely, teacher-rated child problem behaviors were significantly negatively correlated with overall engagement (*r* = −0.55, *p* = 0.01), engagement with the activity/materials (*r* = −0.56, *p* = 0.01), and with goals (*r* = −0.76, *p* < 0.001). Again, the correlation with the engagement with peers dimension was not statistically significant, but was negative (*r* = −0.26, *p* = 0.32). Overall, these analyses revealed interesting insights important for future iterative development of the intervention. First, although previous work found that the Block Play Intervention was particularly efficacious for children whose parents had less educational attainment (Schmitt et al., 2018a) we found that children whose parents have higher educational attainment were more actively engaged with the intervention than their peers. This finding highlights a need to unpack potential reasons and explore additional supports for increasing active child engagement in future iterations as we strive to create equitable interventions appropriate for children from diverse backgrounds. We also found that children who were rated as engaging in more problem behaviors in the classroom by their teacher were less engaged with the intervention, and particularly with the learning goal. Again, in future iterations of the Block Play Intervention, we hope to explore how to scaffold these children’s goal-orientation.

##### 3.1.4.2 Linking active child engagement to outcome measures in the Block Play Intervention

Next, we moved to considering how children’s overall active engagement and engagement with the activity/materials, peers, and learning goals in the Block Play Intervention related to gains in self-regulation and math outcomes. We calculated pre-to post-gain scores in three outcomes of interest: behavioral self-regulation, measured by the Head-Toes-Knees-Shoulders task (HTKS; McClelland et al., 2014); cognitive flexibility, measured by the Three-Dimensional Change Card Sort Task (DCCS; Zelazo, 2006); and mathematics-specific language, measured by the researcher-developed Math Language Assessment (Purpura and Logan, 2015). See Schmitt et al. (2018a) for assessment details. We estimated correlations among these gain scores and children’s active engagement with the activity/materials, peers, and learning goals, as well as overall engagement (see Table 2). Four children were missing post-test data on these measures. None of the dimensions of engagement or overall engagement were significantly correlated with the gain scores (all *p* values > 0.05). Despite this, the pattern of correlation coefficients revealed interesting associations among active child engagement and growth in the outcome variables. For example, overall engagement with the intervention was similarly related to gains in behavioral self-regulation (*r* = 0.20, *p* = 0.39) and cognitive flexibility (*r* = 0.19, *p* = 0.44). However, when the dimensions of engagement variables were considered separately, gains in behavioral self-regulation were similarly related to engagement with the activity/materials (*r* = 0.18, *p* = 0.44), peers (*r* = 0.19, *p* = 0.43), and goals (*r* = 0.17, *p* = 0.49), but gains in cognitive flexibility were more related to active child engagement with peers (*r* = 0.39, *p* = 0.09) than with the activity/materials (*r* = 0.06, *p* = 0.71) or goals dimensions (*r* = −0.03, *p* = 0.91). Gains in mathematics-specific language may also be more related to one dimension of engagement with the intervention (activity/materials, *r* = 0.22, *p* = 0.35) than the others (peers, *r* = 0.08, *p* = 0.75; goals, *r* = −0.04, *p* = 0.74). Although these correlation analyses are underpowered, the pattern of results supports our theoretical assumptions that active child engagement with an intervention is multi-dimensional, and engagement with specific dimensions may be particularly important for supporting different outcomes.

In larger samples, along with traditional measures of fidelity (e.g., dosage and adherence) the separate dimensions of engagement should be considered as moderators of treatment effects on outcome measures. For example, in future iterations of the Block Play Intervention, we plan to test dimensions of engagement as moderators of the effect of condition. We will also test interactions among these dimensions and hypothesize that children who are highly engaged across dimensions will benefit the most from the intervention.

## 4. Conclusion and future directions

Despite theoretical models that include multiple factors of participant responsiveness, the crucial aspect of active participant engagement is often overlooked, especially for young child participants. Some early SEL interventions (e.g., Nesbitt and Farran, 2021) have mixed evidence of efficacy but measuring active engagement with an intervention at the child level may help the field to unpack these mixed results. Furthermore, the multi-dimensionality and variability of children’s active engagement with the intervention must be considered to capture children’s experiences in the complex intervention environment. In this article, we have presented a four-step protocol for identifying, operationalizing, and analyzing children’s active engagement with multiple dimensions of an intervention. We encourage fellow researchers to prioritize incorporating participant engagement at the child level into the measurement of implementation fidelity in SEL interventions with young children and conclude by presenting suggestions to further this work. Although this protocol has been initially applied to a pilot study of a researcher-implemented intervention with a small sample size, we believe it can be scaled for use with larger and more comprehensive intervention studies.

In the fourth step of the protocol, we suggested that future work using this protocol may wish to explore contextual factors that predict the dimensions of engagement. For example, research specifically focused on supporting SEL through play-based learning would benefit from the examination of play as it is defined within different cultural contexts. Children’s play-based learning experiences are scaffolded by their culture and given the impact of globalization, successful interventions would require consideration of the different cultural belief systems of the children and families included in the study (Harkness and Super, 1993). Children have different exposure to play activities within the home environment which affects the variety of play experiences that children are exposed to and possibly their level of active engagement in various dimensions of an intervention activity. Future work is also needed to consider how individual children’s engagement may affect peers’ engagement, especially during group work.

Our example, the Block Play Intervention, did not include an active control condition, but researchers that include a closely aligned active control condition may wish to code active child engagement with the active control condition. For example, in another iteration of the Block Play Intervention, we plan to test the efficacy of the semi-structured play sessions against a free block play condition, in which children are not given specific building goals. We will code and compare engagement with the activity and with peers to compare to the semi-structured condition to test whether there are differences in engagement by condition and if individual differences in engagement relate to gains in the target outcomes.

We focused this article on child participants’ engagement in interventions, but future work is needed to consider the bi-directional relations between interventionist and child-directed factors of implementation. For example, theoretical models posit that interventionist-directed behaviors influence participants’ responsiveness to an intervention (e.g., a cascade model; Berkel et al., 2018). However, child engagement across the dimensions likely also influences interventionist behaviors (Berkel et al., 2011). That is, interventionist adherence to a script and quality of delivery is likely influenced by children’s engagement in the activity at hand, as the interventionist responds to the children’s behavior. The Block Play Intervention was a researcher-implemented intervention, but researchers testing teacher-implemented SEL interventions may also include measures of active teacher participant engagement to consider relations among teacher and child engagement variables. Untangling and determining the best ways to capture the interaction between these factors is an important next step for applying integrated theoretical models of program implementation to practice.

Finally, protocols are also needed to guide the nuanced measurement and analysis of other understudied aspects of fidelity. For example, quality of the delivery of an intervention also includes multiple dimensions and is varied between and within interventionists. Intervention work with school-aged children has explored some of the dimensions of this domain (e.g., practice opportunities, modeling, feedback, scaffolding; Doabler et al., 2021). Additional work is needed to unpack quality in interventions designed for young children and to create general protocols for guiding the identification, operationalization, and analyses of these variables.

In summary, we hope to encourage SEL intervention researchers to consider active child engagement as a worthwhile area of focus in the measurement of implementation fidelity. We have introduced the four-step protocol as a general guide for capturing dimensions of active child engagement. In doing so, the field may be better able to discover which early SEL interventions work, under which conditions, and for whom.

## Data availability statement

The raw data supporting the conclusions of this article will be made available by the authors, without undue reservation.

## Ethics statement

The studies involving human participants were reviewed and approved by Purdue University Institutional Review Board. Written informed consent to participate in this study was provided by the participants’ legal guardian/next of kin.

## Author contributions

BD conceptualized the protocol, conducted analyses, interpretation of data, and led writing of manuscript. BD, TP, EG, and LB wrote the manuscript. TZ, IK, and KM conducted data coding and analysis. SS conceived of original study, directed data collection, provided input on conceptualization of protocol, and interpretation of data. All authors provided critical feedback and approved the submitted version.
