# Corrigendum: Moving beyond dosage and adherence: a protocol for capturing dimensions of active child engagement as a measure of fidelity for social-emotional learning interventions

**DOI:** 10.3389/fpsyg.2023.1261076

**Published:** 2023-08-01

**Authors:** Brianna L. Devlin, Tanya M. Paes, Elyssa A. Geer, Lindsey M. Bryant, Tracy M. Zehner, Irem Korucu, Kathleen Morse, Robert J. Duncan, David J. Purpura, Sara A. Schmitt

**Affiliations:** ^1^Human Development and Family Studies, Purdue University, West Lafayette, IN, United States; ^2^Yale Center for Emotional Intelligence, School of Medicine, Yale University, New Haven, CT, United States

**Keywords:** social-emotional learning (SEL), social-emotional learning interventions, implementation, child engagement, participant responsiveness, preschool

In the published article, the funding statement was omitted. The correct funding statement appears below.

